# Paralysis of Side of Face—Treated by Operation

**Published:** 1871-02

**Authors:** J. E. Garretson


### ARTICLE VIII.

### *Paralysis of Side of Face—Treated by Operation.*

By J. E. Garretson, M. D.

The following case is recorded simply as an experience which may be useful as a reference :

The lady applied two years back for relief from the deformity exhibited, the irregularity of features being dependent on injury done the facial nerve in an inflammation and necrosis of the temporal bone from accident met with in childhood ; paralysis was complete ; not the slightest power existed in the muscles of the affected side.

The desire of patient was for a symmetrical mouth and face ; the question was, the accomplishment of such an end.

Treatment.—A case of this kind treated by any of the various means of nerve stimulation is, of course, a consideration not involved. What is done can only be from the mechanical stand-point. The indications being three fold :

1st. To reduce the flabby redundancy of the paralyzed cheek.

2nd. To give comlineess and regularity to the mouth.

3d. To antagonise the muscular action (when in play) of the vital side.

In this case these indications were attempted to be met by means as follows: A study was made of the cheek, and what was deemed to be the redundant tissue was included in an ellipse drawn with a lead-pencil, the apices being, the one, at the middle of the nose, the other, at the angle of the lower jaw. Such direction of the ellipse being with a purpose of raising the angle of the mouth. Satisfied that such removal would be found rightly placed to meet the two first indications, the part was cut out. In the operation the facial artery was the only vessels needing a ligature, and even this, indeed, ejected no more blood than does an ordinary coronary.

To bring the parts together, three hare-lip pins were used, and agreeably to my surprise, so direct and immediate was the union that it was found permissable to dispense with two of them on the following day ; the third, the middle one, was left in until the fourth day ; but this, not seemingly from any necessity. The ligature, a strand of ligature silk, remained firm for three weeks, and was finally taken away only by the use of a traction quite as great, I should think, as would have sufficed for its removal the moment after it was placed about the vessel.

The result of this procedure was very satisfactory. With the features in a state of rest, nothing more, it would seem, could be desired.

The third indication, however, showed itself a most important one. Emotion of any kind altered this mechanical harmony of the parts and exhibited the non-vitality of the side operated upon ; that is to say, that in laughter, for example, the superior and lateral levators would pull up the well angle, with no corresponding action on the diseased side. This was, of course, a matter which had been originally considered. The indication was met with remarkable success, as follows: A piece of rubber tubing four inches long possessed of an elastic power, adapted to the requirements of the case, was attached by one of its ends to a hair-pin (the ordinary pin used by ladies in dressing the hair). To its other end was united a piece of strong but delicate gill-net string, and this, in turn was connected with a small strip of flesh-colored court-plaster.

The application of this piece of mechanism—an artificial muscle, let us call it—was made as follows: The plaster was softened and applied to the dead side of the face, as far back upon the cheek as would answer the purpose. When fixed, the lady, standing before her glass, would excite the displacing muscles into play, and antagonize them by drawing slightly backward the dead side with the artificial muscle, and fix it in such required tension by the pin fixed into one of the coils of her hair, the rubber lying entirely concealed by such coil. When applied, only the plaster could be seen, the string being hid by the hair.

This rubber muscle answered its purpose admirably The fear that the plaster would irritate, and, perhaps, ulcerate the skin seems to have been without foundation. At any rate this held good for six months of use, which was as long as I was able to have oversight of the case, the lady living in a distant city. Should, however, this accident have supervened, it was evident, after a very few days of experience, that a fashion or habit might soon be attained of accomplishing the same object by the use of the fingers applied in such common manner as not to elicit attention.

The result of this operation, the only one of the kind I have performed, has given results which I think will warrant its repetition.—*Medical and Surgical Reporter.*

